# COVID-19 vaccine effectiveness against symptomatic SARS-CoV-2 infections, COVID-19 related hospitalizations and deaths, among individuals aged ≥65 years in Portugal: A cohort study based on data-linkage of national registries February-September 2021

**DOI:** 10.1371/journal.pone.0274008

**Published:** 2022-09-13

**Authors:** Ausenda Machado, Irina Kislaya, Ana Paula Rodrigues, Duarte Sequeira, João Lima, Camila Cruz, Pedro Pinto Leite, Carlos Matias Dias, Baltazar Nunes

**Affiliations:** 1 Departamento de Epidemiologia, Instituto Nacional de Saúde Doutor Ricardo Jorge, Lisbon, Portugal; 2 Escola Nacional de Saúde Pública (ENSP/NOVA), Centro de Investigação em Saúde Pública (CISP/NOVA), Universidade NOVA de Lisboa, Lisbon, Portugal; 3 Serviços Partilhados do Ministério da Saúde, Lisbon, Portugal; 4 Direção de Serviços de informação e análise,- Direção Geral da Saúde, Lisbon, Portugal; National Center for Global Health and Medicine, JAPAN

## Abstract

**Background:**

Using data from electronic health registries, this study intended to estimate the COVID-19 vaccine effectiveness (VE) in the population aged 65 years and more, against symptomatic infection, COVID-19-related hospitalizations, and deaths, overall and by time since complete vaccination for the period February to September 2021

**Methods:**

We established a cohort of individuals aged 65 and more years old, resident in Portugal mainland, using the National Health Service User number to link eight electronic health registries. Outcomes included were symptomatic SARS-CoV-2 infections, COVID-19-related hospitalizations or deaths. The exposures of interest were the mRNA vaccines (Comirnaty or Spikevax) and the viral vector (Vaxzevria) vaccine. Complete schedule VE was estimated as one minus the confounder adjusted hazard ratio, for each outcome, estimated by time-dependent Cox regression with time-dependent vaccine exposure.

**Results:**

For the cohort of individuals aged 65–79 years, complete scheme VE against symptomatic infection varied 43 (95%CI: 37–49) (Vaxzevria) and 65 (95%CI: 62–68) (mRNA vaccines). This estimate was slightly lower in the ≥80 years cohort (53, 95%CI: 45–60) for mRNA vaccines). VE against COVID-19 hospitalization varied between 89% (95%CI: 52–94) for Vaxzevria and 95% (95%CI: 93–97) for mRNA vaccines for the cohort aged 65–79 years and was 76% (95%CI: 67–83) for mRNA vaccines in the ≥80 years cohort. High VE against COVID-19-related deaths was estimated, for both vaccine types, 95% and 81 (95%CI:76–86) for the 65–79 years and the ≥80 years cohort, respectively. We observed a significant waning of VE against symptomatic infection, with VE estimates reaching approximately 34% for both vaccine types and cohorts. Significant waning was observed for the COVID-19 hospitalizations in the ≥80 years cohort (decay from 83% (95%CI:68 to 91) 14–41 days to 63% (95%CI:37 to 78) 124 days after mRNA second dose). No significant waning effect was observed for COVID-19-related deaths in the period of follow-up of either cohort.

**Conclusions:**

In a population with a high risk of SARS-CoV-2 complications, we observed higher overall VE estimates against more severe outcomes for both age cohorts when compared to symptomatic infections. Considering the analysis of VE according to time since complete vaccination, the results showed a waning effect for both age cohorts in symptomatic infection and COVID-19 hospitalization for the 80 and more years cohort.

## Introduction

Vaccination is an important tool to control the COVID-19 pandemic. In Portugal COVID-19 vaccines started to be deployed on December 27, 2020, prioritising individuals with a higher risk of exposure and/or COVID-19 complications [1]. On February 3, 2021,mass vaccination was implemented starting with those aged 80 or more years and then by decreasing age groups [1]. The first vaccine to be approved and administered was mRNA Pfizer/BioNTech (Comirnaty) (21/12/2020), followed by Moderna (Spikevax) (06/01/2021), AstraZeneca (Vaxzevria) (29/01/2021) and Janssen vaccine (COVID-19 Vaccine Janssen) (11/03/2021) [2].

The Vaxzevria vaccine was first given to those aged less than 65 years [3], but the recommendation changed in April 2021, to be administrated to those aged more than 60 years old [4]. A single-dose Janssen vaccine was recommended to all males aged 18 and more years old and females aged 50 or more years [5]. In early October 2021, vaccine coverage in the Portuguese population reached a milestone of 84% (complete schedule) and was estimated to be higher than 95% for those aged 65 and more years [6].

Coinciding with the start of the vaccination campaign, Portugal faced the third wave of COVID-19. In January 2021, the 14-day incidence rate reached the peak of 1,667 cases per 100,000 inhabitants and the transmissibility (Rt) was over 1 [7]. Given the health care rupture, and to control the epidemic, a lockdown was implemented on January 15^th^, 2021. At this point, the predominant circulating variant was the B.1.7.1 (alpha variant), which was progressively replaced by the B.1.617.2 (delta variant) that became predominant in June 2021. Data on the variants of concern (VOC) surveillance system indicate that by the end of May 2021 the proportion of circulating variants were 87.7% and 4.8%, for alpha and delta variants respectively [8]. By the end of June 2021, these figures were 9.8% and 88.2%, for alpha and delta variants, respectively [9]. Along with the end of the lockdown and the increasing frequency of the Delta variant, an increasing trend of the epidemic activity was observed in all Portuguese regions affecting mainly young adults and adolescents [7]. There were several changes in the national testing strategy over time, but until October 13^th^, 2021, the recommendation was still in place to test vaccinated persons in the same circumstances as for non-vaccinated (suspected or contact with COVID-19, regular testing in high risk of exposure or high-risk populations, regular testing in specific settings such schools and workplaces).

Post-licencing COVID-19 vaccine studies are needed to measure the protection provided by vaccination in real-world conditions. They add to the information provided by clinical trials, as larger and more heterogeneous populations are included. Early evaluations focused mainly on the performance of Comirnaty, and Vaxzevria adenoviral vaccines, showing high short-term effectiveness against SARS-CoV-2 infections and severe outcomes in the general population and older adults [10, 11]. VE against COVID-19-related hospital admissions and mortality in older adults populations varied between 94–97% for the completed vaccination schedule in December 2020—April 2021 [10–13]. Following the emergence of the Delta SARS-CoV-2 VOC, studies showed a decrease in VE for both mRNA and Vaxzevria vaccines for symptomatic infections in the general population [14–16], but sustained VE estimates against hospitalizations [14–17] and COVID-19-related mortality [15].

Immunological studies suggest a decrease of IgG and neutralizing antibody titers 3–6 months after completion of the two-dose schedule [18, 19]. Continued monitoring of VE over time is important to provide input on a potential vaccine waning effect, as has been observed with vaccines against other respiratory pathogens [20, 21] and COVID-19 vaccines [16].

In the context of multiple vaccines and variant epidemiological setting, it is important to assess the protection conferred by the COVID-19 vaccines in use. Moreover, monitoring over time allows detection of potential decay of VE and may be important for the decision on the vaccination strategy.

This study aimed to estimate the effectiveness of COVID-19 vaccines against symptomatic infections, COVID-19-related hospitalizations and deaths in Portuguese adults aged 65 years old or more from February-September 2021

## Methods

### Study design

A historical cohort study, based on deterministic data linkage of nationwide electronic health registries [15] was designed to measure the VE of COVID-19 vaccines used in Portugal. Three outcomes were evaluated: i) symptomatic infection, ii) COVID-19 hospitalizations, and iii) COVID-19-related deaths in individuals aged 65 or more years old (yo).

Data extraction and linkage were performed on 16th September 2021. The National Health Service User (NHSU) individual unique numeric identifier was used to link all databases. These included the NHSU, the national vaccination registry, the National Information System for Epidemiologic Surveillance, the National Death Registry, the Primary Information System, the Primary Care Clinical Monitoring System of COVID-19 Patients in Home Isolated, the National Database of Medicine and Treatment Prescriptions and the National Database of Hospital Discharges.

### Target population and exclusion criteria

The study population comprised Portuguese residents aged 65 or more years old registered in the NHSU database and residing in Portugal mainland, eligible for COVID-19 vaccination during the study period.

Individuals resident in long-term care facilities and other institutions were excluded as the timing for vaccination and potential exposure to SARS-CoV-2 were different from community-dwelling individuals. Given that the use of electronic registries does not allow the proper identification of institutionalized individuals, the cohort start was set when the vaccination coverage in the population considered a priority for vaccination was higher than 80%, which also includes the population living in long-term facilities.

The study was also restricted to those aged less than 110 years and to those with no prior SARS-CoV-2 infection. For those aged 65 to 79 years old, to ensure that the cohort included individuals with the same likelihood to be vaccinated, only the subset of frequent users of the national health service [22], i.e., individuals who had at least one contact with a primary health care unit in the previous 3 years, were included in the analysis. Finally, for the 80 and more years population, the cohort was also restricted to those who received at least one influenza or pneumococcal vaccine in the last 5 years to increase the likelihood of being a current health care user.

### Study period

The observation period was established for each age-group cohort following the vaccination plan rollout. Namely, from 2nd February 2021 for individuals with 80 or more years and 30th of March 2021 for the 65 to 79 years of age. The follow-up was ended in the date of the last observed event in each cohort (date of last observed event in supplementary material).

### Variables

#### Outcomes

An individual was considered as symptomatic infection if she/he had a laboratory-confirmed infection and at least one of the following symptoms: feverishness/ fever, cough, sore throat, dyspnea, anosmia, diarrhea, abdominal pain, fatigue, malaise, myalgia, nausea or vomiting in an interval of <25 to >15 days since laboratory confirmation for SARS-CoV-2 infection notified in the National Information System for Epidemiologic Surveillance or in the Primary Care Clinical Monitoring System of COVID-19 Patients in Home Isolated.

In Portugal, any individual presenting symptoms similar to SARS-CoV-2 infection or that had contact with an infected individual is tested by Nucleic Acid Amplification Tests (TAAN) or Rapid Antigen Test (RAT) if TAAN were not available. Also, all persons having high risk of exposure (eg. Health Care Workers), or at high risk of complications or moving into specific settings (long-term care facilities, schools, workplaces with more than 150 professionals) were regularly tested regardless of their vaccination status until 13 of October 2021. TAAN tests were used in high-risk contexts, and RAT in other settings [23].

For COVID-19 hospitalization, we considered all individuals with a laboratory-confirmed infection that required hospitalization coded at discharge with COVID-19 as the primary diagnosis (ICD10 code U07.1) [24]. Hospitalizations were retrieved through the national database of hospital discharges that contains information on all hospitalizations in public hospitals in mainland Portugal. After discharge, all hospitalizations are coded by trained professionals according to the international classification of disease (ICD), 10^th^ version. Timeliness of codification may vary between regions and hospitals and may take up to 6 months for completion.

A COVID-19-related death was defined as all-cause death with a positive RT-PCR test within the previous 30 days [25].

#### Exposure

Data on COVID-19 vaccine exposure were collected from the national vaccination registry (VACINAS). Participants without documented COVID-19 vaccine uptake were classified as unvaccinated and considered a reference group for the analysis. Participants were classified as having complete vaccination scheme 14 or more days after the second dose of vaccine uptake. Participants who received at least one dose of vaccine but do not fulfil the definition of complete vaccination were classified as with incomplete vaccination and were not covered by the analysis. To evaluate the hypothesis of VE waning, the time after complete vaccination was additionally categorized into 28-day intervals.

#### Confounding factors

Potential confounders included age groups, sex, municipality level European Deprivation Index (EDI) quintile [26], number of chronic diseases(anemia, asthma, cancer, cardiac disease, stroke, dementia, diabetes, hypertension, chronic liver disease, neuromuscular disease, renal disease, rheumatologic disease pulmonary disease, obesity, immunodeficiency, tuberculosis), number of laboratory SARS-CoV-2 tests during 2020, previous influenza or pneumococcal vaccines uptake.

### Statistical methods

Descriptive statistics were used to characterize study participants. VE was estimated by vaccine type (mRNA or Vaxzevria). Given the different vaccination and observational periods, we estimated VE separately for two age-group cohorts, 65–79 and ≥80 years of age.

Observation time was stratified in 7-day intervals from the start to the ending date of the observational periods, in each cohort. Time-dependent person-year exposure experience was calculated for unvaccinated and vaccinated persons according to the number of doses and time of exposure to each dose. Participants who developed one primary outcome or who died during the study period were excluded from the denominator after the event.

For each outcome, incidence rates per 10,000 person years were calculated for unvaccinated exposure and vaccine exposure period, according to the number of doses and time since each dose. Crude incidence rate ratios among each vaccination group and unvaccinated were calculated with their respective 95% confidence intervals.

VE was computed as one minus the confounder adjusted hazard ratio, for each outcome, estimated by time-dependent Cox regression, adjusted for age group, sex, municipality level EDI quintile, number of chronic conditions, number of SARS-CoV-2 tests in 2020, and uptake of influenza or pneumococcal vaccine in previous 3 years of the study period.

For ≥80 years cohort group the hypothesis of VE waning after complete schedule was evaluated by comparing hazard ratio between the group of individuals with 14 to 41 days to those with 124 or more days of exposure. For the 65–79 years cohort due to a shorter follow-up period, the VE waning was assessed up to 98+ days for symptomatic infection and up to 70+ days for hospitalizations and deathAll statistical analysis was performed in R Computing Environment, version 4.0.5.

### Ethical considerations

The study protocol was approved by the Ethical Committee of the Instituto Nacional de Saúde Doutor Ricardo Jorge (INSA) on 19/01/2021 and the data protection officers of INSA and SPMS 002/UPDCS/2021 on 09.04.2021. Given that all data were irreversibly anonymized prior to analysis, the need for consent was waived by the Ethical Committee.

## Results

### Participants

Database extraction on 16^th^ September 2021 contained 2,117,002 individuals aged 65 to 79 years and 923,450 individuals aged ≥80 years. After excluding, according to previously described criteria, cohorts of sizes 1,414,909 aged 65 to 79 years (S1 Fig) and 470,023 aged ≥80 years were constituted (S2 Fig).

Distribution of vaccines varied by age cohort. For the 65 to 79 years cohort, the Corminaty vaccine was administrated to 644,959 individuals (45.6%) and the second most frequent vaccine was from Vaxzevria (499,770 persons, 35.3%). Spikevax (115,217, 8.1%) and Janssen (40,484, 2.9%) were less frequently administered in this age cohort (S1 Fig). The majority of adults aged 80 and more years, were vaccinated with the Corminaty (379,285, 80.7%). The other vaccines were also available for this age group but were less frequently used (Spikevax: 56,037 individuals, 11.9%; Vaxzevria: 8,509 individuals, 1.8%; Janssen: 2,191 individuals, 0.5%) (S2 Fig).

The unvaccinated were older than those vaccinated with Vaxzevria in the 80 and more yo cohort (median of 86 vs 82, respectively). In both cohorts, the proportion of individuals without any chronic condition was higher in the unvaccinated group than in the vaccinated with Vaxzevria or mRNA vaccines (65 to 79 yo cohort: 48.9% vs 22.9% or 29.3% or 22.7%; 80 and more yo cohort: 37.2% vs 11.2% or 14.3% or 10.2%) (S1 and S2 Tables).

During the study period, in the 65 to 79 years cohort, a total of 4,282 symptomatic infections, 338 hospitalizations, and 172 deaths were observed (Table 1), compared to a total of 3,078 symptomatic infections, 854 hospitalizations, and 764 COVID-19-related deaths observed in the 80 and more years cohort (Table 2).

**Table 1:** Symptomatic SARS-CoV-2 infection, COVID-19 related hospitalizations and deaths, incidence, hazard ratios and vaccine effectiveness by mRNA (Comirnatyor Spikevax) and Vaxzevria vaccination status for individuals aged 65–79 years, Portugal, March–September 2021 (n = 1,414,909).

Vaccine Status	Person years	events (n)	Rate per 10,000 py	Confounder-adjusted HR 95% CI	VE 95% CI
**Symptomatic SARS-CoV-2 infection**					
mRNA vaccine (Comirnatyor Spikevax)—Total	361,642	3,185			
Unvaccinated	156,266	1,457	93.2		
**Complete vaccination**	**205,376**	**1,728**	**84.1**	**0.35 (0.32 to 0.38)**	**65 (62 to 68)**
**14–41 days**	**57,355**	**188**	**32.8**	**0.21 (0.17 to 0.24)**	**79 (76 to 83)**
42–69 days	56,258	521	92.6	0.32 (0.29 to 0.36)	68 (64 to 71)
70–97 days	53,145	596	112.1	0.41 (0.37 to 0.47)	59 (53 to 63)
**≥98 days (max = 148)**	**38,618**	**423**	**109.5**	**0.61 (0.52 to 0.71)**	**39 (29 to 48)**
*Waining effect*				2.9 (2.4 to 3.6)	
Vaxzevria–Total	233,648	2,554			
Unvaccinated	156,266	1,457	93.2		
**Complete vaccination**	**77,386**	**1,097**	**141.8**	**0.57 (0.51 to 0.63)**	**43 (37 to 49)**
**14–41 days**	**37,135**	**546**	**147.0**	**0.52 (0.46 to 0.58)**	**48 (42 to 54)**
42–69 days	33,830	495	146.3	0.67 (0.58 to 0.77)	33 (23 to 42)
**≥70 days (max = 142)**	**6,417**	**56**	**87.3**	**0.66 (0.48 to 0.90)**	**34 (10 to 52)**
*Waining effect*				1.27 (0.92 to 1.76)	
**COVID-19 related hospitalizations**					
mRNA vaccine (Comirnaty or Spikevax)–Total	362,147	298			
Unvaccinated	156,564	266	17.0		
**Complete vaccination**	**205,583**	**32**	**1.6**	**0.05 (0.03 to 0.07)**	**95 (93 to 97)**
**14–41 days**	**57,366**	**10**	**1.7**	**0.05 (0.03 to 0.90)**	**95 (90 to 97)**
42–69 days	56,293	9	1.6	0.03 (0.02 to 0.06)	97 (94 to 98)
**≥70 days (max = 148)**	**91,924**	**13**	**1.4**	**0.07 (0.04 to 0.14)**	**93 (86 to 96)**
*Waining effect*				1.35 (0.57 to 3.22)	
Vaxzevria—Total	234,086	286			
Unvaccinated	156,564	266	17.0		
**Complete vaccination**	**77,522**	**20**	**2.6**	**0.11 (0.06 to 0.48)**	**89 (52 to 94)**
**COVID-19 related deaths**					
mRNA vaccine (Comirnaty or Spikevax)—Total	362,210	159			
Unvaccinated	156,623	129	8.2		
**Complete vaccination**	**205,587**	**30**	**1.5**	**0.05 (0.03 to 0.08)**	**95 (92 to 97)**
**14–41 days**	**57,366**	**5**	**0.9**	**0.05 (0.02 to 0.12)**	**95 (88 to 98)**
42–69 days	56,294	7	1.2	0.03 (0.02 to 0.08)	97 (92 to 98)
**≥70 days (max = 148)**	**91,927**	**18**	**2.0**	**0.07 (0.04 to 0.13)**	**93 (87 to 96)**
*Waining effect*				1.34 (0.48 to 3.75)	
Vaxzevria—Total					
Unvaccinated	156,623	129	8.2		
**Complete vaccination**	**77,527**	**13**	**1.7**	**0.05 (0.03 to 0.10)**	**95 (90 to 97)**

**SARS-CoV-2 symptomatic case**: Individual with a SARS-CoV-2 laboratory confirmation during study period and at least one symptom (feverishness/ fever, cough, sore throat, dyspnea, anosmia, diarrhea, abdominal pain, fatigue, malaise, myalgia, nausea or vomiting) declared from in the period -15 to 25 days of laboratory confirmation; Complete vaccination: 2 doses ≥ 14 days; Rate: Per 1,000 person-years; Confounder-adjusted HR: Confounder-adjusted hazard ratio obtained by time-dependent Cox regression with vaccine exposure as time-dependent, adjusted for age group, sex, health region, municipality level European Deprivation quintiles, number of chronic diseases, number of SARS-CoV-2 tests performed in 2021, influenza or pneumococcal vaccine uptake in the past 3 years and time (7-day periods); VE was calculated by (1-HR)*100.

**Table 2:** Symptomatic SARS-CoV-2 infection, COVID-19 related hospitalizations and deaths, incidence, hazard ratios and vaccine effectiveness by mRNA (Comirnaty or Spikevax) vaccination status for individuals aged ≥80 years, Portugal, February–September 2021 (n = 470,023).

Vaccine Status	Person years	events (n)	Rate per 10,000 py	Confounder-adjusted HR 95% CI	VE 95% CI
**Symptomatic SARS-CoV-2 infection**					
*mRNA vaccine (Comirnaty or Spikevax)–Total*	230,371	3,078			
Unvaccinated	61,194	1,690	27.6		
**Complete vaccination**	**169,177**	**1,388**	**82.0**	**0.47 (0.41 to 0.55)**	**53 (45 to 60)**
**14–41 days**	**32,731**	**53**	**16.2**	**0.28 (0.21 to 0.39)**	**72 (61 to 79)**
42–69 days	32,457	97	29.9	0.36 (0.28 to 0.47)	64 (53 to 72)
70–97 days	32,036	258	80.5	0.47 (0.38 to 0.57)	53 (43 to 62)
98–123 days	29,101	353	121.3	0.50 (0.41 to 0.60)	50 (40 to 59)
**≥124 days (max = 203)**	**42,852**	**627**	**146.3**	**0.66 (0.52 to 0.71)**	**34 (29 to 48)**
*Waining effect*				2.33 (1.68 to 3.22)	
**COVID-19 related hospitalizations**					
*mRNA vaccine (Comirnaty or Spikevax)–Total*	230,967	**854**			
Unvaccinated	61,607	723	117.4		
**Complete vaccination**	**169,360**	**131**	**7.7**	**0.24 (0.17 to 0.33)**	**76 (67 to 83)**
**14–41 days**	**32,740**	**11**	**3.4**	**0.17 (0.09 to 0.32)**	**83 (68 to 91)**
42–69 days	32,469	15	4.6	0.19 (0.10 to 0.34)	81 (66 to 90)
70–97 days	32,057	37	11.5	0.26 (0.16 to 0.40)	74 (60 to 84)
98–123 days	29,138	36	12.4	0.26 (0.17 to 0.42)	74 (58 to 83)
**≥124 days (max = 203)**	**42,956**	**32**	**7.4**	**0.27 (0.63 to 0.22)**	**63 (37 to 78)**
*Waining effect*				2.21 (1.00 to 4.90)	
**COVID-19 related deaths**					
*mRNA vaccine (Comirnaty or Spikevax)–Total*					
Unvaccinated	61,790	575	93.1		
**Complete vaccination**				**0.19 (0.14 to 0.24)**	**81 (76 to 86)**
**14–41 days**	**32,742**	**9**	**2.7**	**0.13 (0.07 to 0.29)**	**87 (71 to 93)**
42–69 days	32,472	12	3.7	0.12 (0.06 to 0.22)	88 (78 to 94)
70–97 days	32,060	30	9.7	0.14 (0.09 to 0.22)	86 (78 to 91)
98–123 days	29,142	56	19.2	0.20 (0.14 to 0.29)	80 (71 to 86)
**≥124 days (max = 203)**	**42,965**	**82**	**19.1**	**0.25 (0.18 to 0.36)**	**75 (64 to 82)**
*Waining effect*				1.75 (0.82 to 3.72)	

**SARS-CoV-2 symptomatic case**: Individual with a SARS-CoV-2 laboratory confirmation during study period and at least one symptom (feverishness/ fever, cough, sore throat, dyspnea, anosmia, diarrhea, abdominal pain, fatigue, malaise, myalgia, nausea or vomiting) declared from in the period -15 to 25 days of laboratory confirmation; Complete vaccination: 2 doses ≥ 14 days; Rate: Per 10,000 person-years; Confounder-adjusted HR: Confounder-adjusted hazard ratio obtained by time-dependent Cox regression with vaccine exposure as time-dependent, adjusted for age group, sex, health region, municipality level European Deprivation quintiles, number of chronic diseases, number of SARS-CoV-2 tests performed in 2021, influenza or pneumococcal vaccine uptake in the past 3 years and time (7-day periods); VE was calculated by (1-HR)*100.

### Vaccine effectiveness against symptomatic infection

mRNA VE against symptomatic infection varied between 53% (95%CI: 45 to 60) in the 80 and more years and 65% (95%CI: 62 to 68) in the 65–79 years cohort. Considering the Vaxzevria vaccine, the VE estimate was 43% (95%CI: 37 to 49) in the 65–79 yo cohort.

Analysis of VE according to time since complete vaccination showed a reduction for mRNA vaccines. The decline was observed in the 80 and more years cohort, (hazard ratio of symptomatic infection of 2.33, 95%CI: 1.68 to 3.22), with VE estimates decreasing from 72% (61 to 79) (14 to 41 days after complete vaccination) to 34% (29 to 48) (124 to 203 days after complete vaccination). For the cohort of individuals aged between 65 to 79 years, the VE estimates declined from 79% (76 to 83) at 14 to 41 days after the second dose, to 39% (29 to 48) at 98 and more days since the second dose, corresponding to a hazard ratio of 2.9 (95%CI: 2.4 to 3.6) (Table 1)

### Vaccine effectiveness against hospitalizations

VE against severe outcomes was higher when compared to symptomatic infection. For the 65–79 years cohort, VE varied between 89% (52 to 94) for Vaxzevria vaccines and 95% (93 to 97) for mRNA vaccines. Considering the 80 and more yeas cohort, mRNA VE against hospitalizations was estimated at 76% (67 to 83). Due to the small size of the group exposed to the Vaxzevria vaccine among 80 or more years old the Vaxzevria VE against hospitalizations was not estimated.

Considering time since vaccination, for the 80 years of age or more cohort, mRNA VE against COVID-19 hospitalization was83% (68 to 91) (14 to 41 days after complete vaccination) and 63% (37 to 78) (124 or more days from complete vaccination). These estimates translated into a decrease in protection between the two periods, with a 2.21 (95% CI 1.0 to 4.9) hazard rate ratio of COVID-19-related hospitalization.

For the 65 to 79 years cohort, no significant VE decay over time was observed.

### Vaccine effectiveness against COVID-19-related deaths

For the mRNA vaccines, VE estimates varied between 81% (76 to 86) in the 80 and more yo cohort and 95% (92 to 97) in the 65- to 79 yo cohort. For the Vaxzevria vaccine, VE against COVID-19-related deaths was estimated at 95% (90 to 97) in the 65–79 years old group.

For both cohorts, no significant VE decay over time was observed.

## Discussion

Main results indicate that VE varied according to the outcome and vaccine type. VE estimates against symptomatic infections range from 43% to 65% in the 65–79 years cohort and 53% in the 80 and more years cohort. These estimates were lower than VE estimated against severe COVID-19 outcomes–hospitalization and death (higher than 89% in the 65–79 years cohort and higher than 76% in the 80 and more years cohort). These results suggest a high level of vaccine protection against severe COVID-19 outcomes in the 65 and more years population that is more prone to COVID-19 related complications.

Compared to other studies we found some differences. A living review, which included studies published up to 25 August 2021, allowed a meta-analysis and indicated that VE against symptomatic infection was 75.7% (95%CI: 69.3 to 80.8) [27]. The differences between our overall VE estimates and other studies might be explained by different study periods and consequently different epidemiological and virological contexts.

Our study comprises a period of high incidence observed at the beginning of the vaccination campaign, corresponding to the third COVID-19 peak in January-February 2021 (for the 80 and more years cohort), but also the fourth wave associated with the circulation of SARS-CoV-2 Delta variant during May-September 2021. As described in the living review, when estimating VE comparing Delta vs Alpha, estimates tended to be 10 to 20% lower for less severe COVID-19 infection but not different for severe outcomes [27].

Considering VE against COVID-19 related death, we could find fewer studies that focused on this severe outcome, as the majority reported VE against severe outcomes which included hospitalizations or death and thus are not comparable with our results [14, 25]. Results from one study conducted in the population aged 60 or more years in Scotland, reported high VE against of 90% (95% CI: 84 to 94) for Vaxzevria vaccine and 87% (95% CI: 77 to 93) for Corminaty vaccine [28]. Our VE estimates against death are in line with these estimates and also support high protection conferred by both vaccines when considering this fatal outcome.

For the 60–79 years cohort, we were able to estimate VE for the different outcomes and two types of vaccines: mRNA (Comirnatyand Spikevax) and adenovirus-based vaccine (Vaxzevria). A similar analysis was not possible for the 80 and more cohort or the Janssen vaccine, as the proportion of vaccinated individuals was too low. For the 65–79 years cohort and considering symptomatic infection, VE estimates indicate lower protection conferred by the adenovirus vaccine (Vaxzveria VE of 43%; 95%CI 37 to 49) vs mRNA (Comirnaty or Spikevax) VE of 65% with 95%CI of 62 to 68. Other brands or type-specific vaccine studies have also reported similar lower VE against symptomatic infection of Vaxzevria when compared to Corminaty vaccine [29, 30].

After complete vaccination schedule, we observed a decay of the VE with time, particularly against symptomatic infection. To highlight that we observed a decaying the 80 and more years cohort, where VE waining was observed for both symptomatic infection and hospitalizations.

The drop over time of VE against symptomatic infection has also been reported by several authors [14–16] but the decay of VE against hospitalization was not reported in that period [14–17]. In part, this result may be explained by an insufficient time of follow-up. Our results indicate a significant decay of VE against hospitalizations in the 80 and more years cohort, from 83% (the peak achieved 14–41 days after complete vaccination) to 63% (after 124 days of complete vaccination).

One valid argument is that this decay may be attributable to the increased circulation of the Delta variant which could have increased the effect on the older aged cohort. However, waning of vaccine effect with time cannot be ruled out. As observed by Tartof et al. [16], the decay on VE against symptomatic infection was observed in all population (even in younger-aged cohorts with less than <45 years old) and this pattern was observed even before the circulation of Delta variant. This study was not focused on VE against specific variants and as such, this hypothesis needs confirmation.

The present study has some limitations, namely related to the datasets used and their quality. For instance, the main dataset used to link data was the NHSU, which contains the unique health number attributed to each individual residing in Portugal. However, the NHSU database could have update issues, and occasional and temporary registrations of NHS users, such as immigrants and asylum seekers, that could artificially increase the number of individuals in a given cohort. To overcome this limitation, several restriction criteria were included, and from the initial extraction, a total of 1,178,975 registries were excluded from the analysis. Final cohort age and sex distribution were comparable to National Statistics estimates for individuals aged 65 and more years [31]. In terms of the prevalence of chronic conditions, final cohorts were also comparable to National estimates (INS 2019).

Another potential limitation is related to information bias. Differential testing guidelines between vaccinated and unvaccinated may affect the opportunity of infection ascertainment in vaccinated and unvaccinated. In Portugal, during the study period, testing guidelines were not different for vaccinated and non-vaccinated individuals. Nevertheless, we cannot rule out the hypothesis that vaccinated individuals could have had a differential (due to acceptance) testing performance if asymptomatic or if they had any contact with the COVID-19 case. Under this hypothesis, there is a potential bias due to different infection ascertainment in vaccinated versus unvaccinated, which might have contributed to overestimating VE against infection. This potential bias is less probable for severe outcomes such as hospitalization. Nevertheless, when we compared the frequency of testing during 2021 between the groups compared in this study we did not observe relevant differences.

In what concerns the COVID-19 hospitalizations outcome, the delay in the update of information in the registries regarding discharge diagnosis might contribute to underrepresenting this specific outcome. Although we didn´t find any reason for having a different delay in the registry of the discharge information between vaccinated and non-vaccinated, there is a possibility of a differential bias if vaccinated had a shorter hospitalization stay when compared to non-vaccinated.

Finally, there is potential for residual confounding bias. Adjustment for confounding was made for several variables, most of them very relevant from a clinical and epidemiological point of view. Nevertheless, residual confounding bias could remain mainly due to the absence of information on non-pharmaceutical protective measures such as the use of facemask, hand washing, and social distancing, all of which could be associated with both vaccine uptake and risk of infection.

Besides the impossibility of excluding the presence of selection or information bias driven by health-seeking behavior, our results are generally in line with results reported in studies performed elsewhere. Finally, as our cohort comprises all the Portuguese NHS users older adults population, we consider our results to represent a description of the direct protection conferred by the COVID-19 vaccines used within the target population, and a good approach to monitoring VE along time and waning of the vaccine protection.

## Supporting information

S1 Fig Selection flowchart 65–79 years old cohort. (PDF)

S2 Fig Selection flowchart 80 or more years old cohort. (PDF)

S1 Table Demographic characteristics and vaccine status of cohort individuals aged 65 to 79 years old, Portugal, March–August 2021 (n = 1,414,909). (PDF)

S2 Table Demographic characteristics and vaccine status of cohort individuals aged 80 or more years old, Portugal, February–August 2021 (n = 470,025). (PDF)
