# Supplementary figures and images for: COVID-19 vaccine effectiveness against symptomatic SARS-CoV-2 infections, COVID-19 related hospitalizations and deaths, among individuals aged ≥65 years in Portugal: A cohort study based on data-linkage of national registries February-September 2021
Source: PLoS One. 2022 Sep 13;17(9):e0274008. doi: 10.1371/journal.pone.0274008 (PMC9469958; doi:10.1371/journal.pone.0274008)

S1 Fig. Selection flowchart 65-79 years old cohort

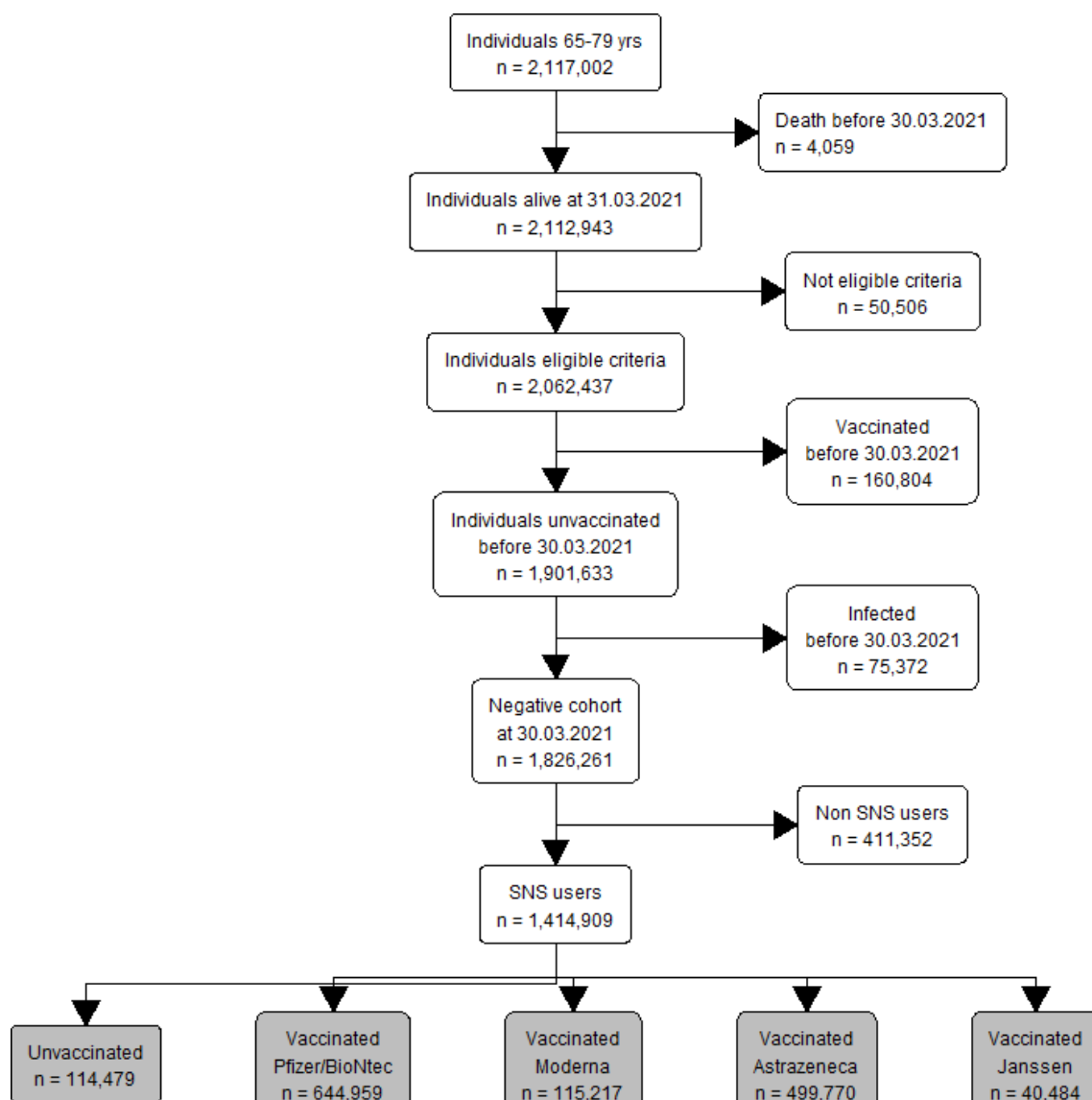

Supplement: S1 Fig — (PDF) [file pone.0274008.s001.pdf]

S2 Fig. Selection flowchart 80 or more years old cohort

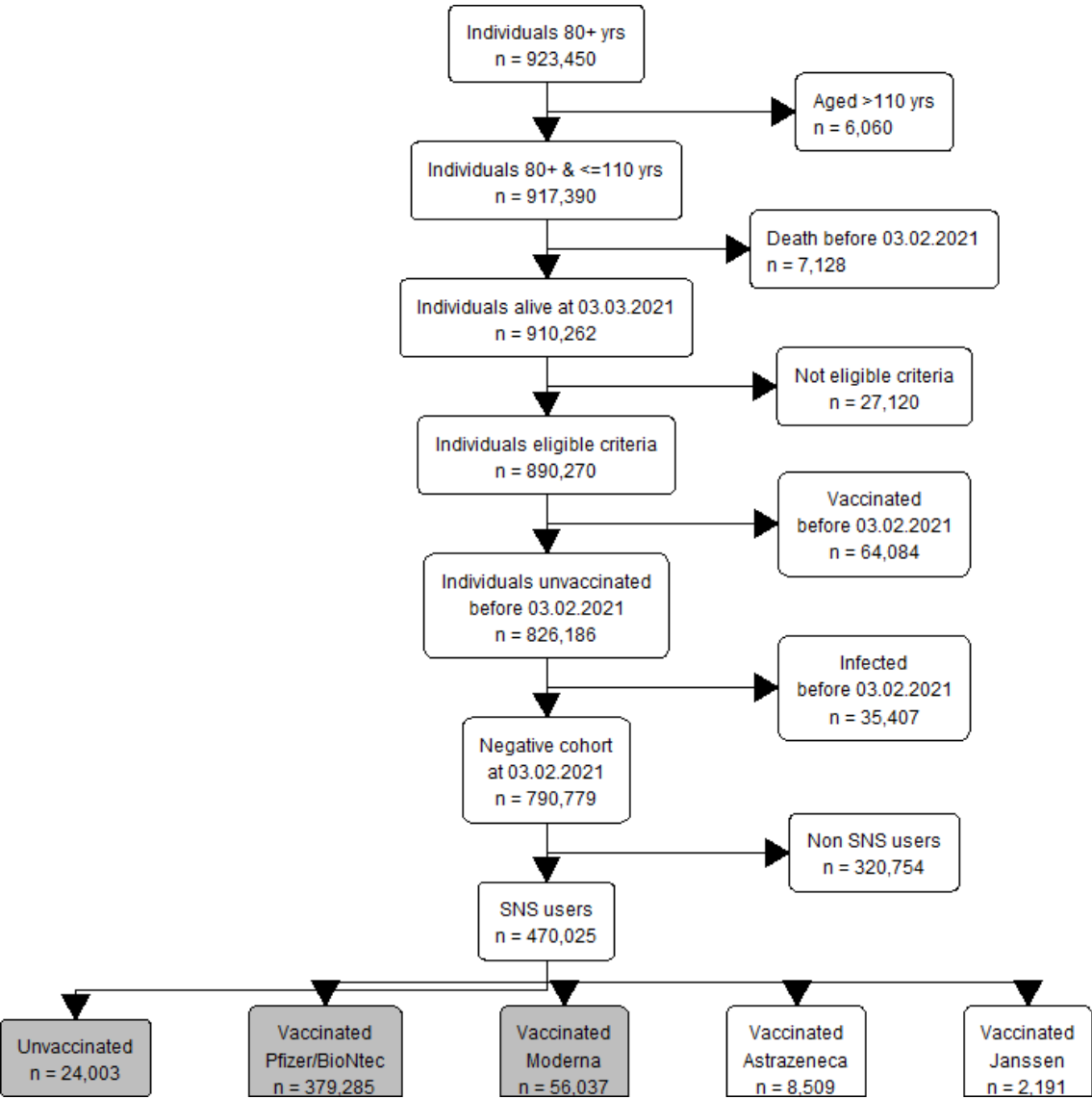

Supplement: S2 Fig — (PDF) [file pone.0274008.s002.pdf]
