# Supplementary material for: COVID-19 vaccine effectiveness against symptomatic SARS-CoV-2 infections, COVID-19 related hospitalizations and deaths, among individuals aged ≥65 years in Portugal: A cohort study based on data-linkage of national registries February-September 2021
Source: PLoS One. 2022 Sep 13;17(9):e0274008. doi: 10.1371/journal.pone.0274008 (PMC9469958; doi:10.1371/journal.pone.0274008)
Supplement: S1 Table — (PDF) [file pone.0274008.s003.pdf]

S1 Table. Demographic characteristics and vaccine status of cohort individuals aged 65 to 79 years old, Portugal, March–August 2021 (n = 1,414,909).

	Vaxzevria 499,770	Janssen 40,484	mRNA 760,176	Unvaccinated 114,479
Age (years), median (IQR)	71.0 (68.0 - 75.0)	69.0 (67.0 - 72.0)	71.0 (68.0 - 75.0)	71.0 (68.0 - 75.0)
Age group, (%)				
65-69	178,573 (35.7%)	21,890 (54.1%)	297,449 (39.1%)	43,262 (37.8%)
70-74	194,191 (38.9%)	12,776 (31.6%)	257,681 (33.9%)	39,124 (34.2%)
75-79	127,006 (25.4%)	5,818 (14.4%)	205,046 (27.0%)	32,093 (28.0%)
Sex Male, n (%)	221,369 (44.3%)	18,407 (45.5%)	332,106 (43.7%)	51,302 (44.8%)
Region, n (%)				
Alentejo	27,817 (5.6%)	2,592 (6.4%)	35,331 (4.6%)	4,538 (4.0%)
Algarve	27,567 (5.5%)	6,318 (15.6%)	29,480 (3.9%)	12,502 (10.9%)
Centro	97,820 (19.6%)	10,582 (26.1%)	133,943 (17.6%)	19,034 (16.6%)
LVT	212,153 (42.5%)	11,646 (28.8%)	234,669 (30.9%)	41,354 (36.1%)
Norte	132,245 (26.5%)	8,864 (21.9%)	322,412 (42.4%)	25,612 (22.4%)
Missing	2,168 (0.4%)	482 (1.2%)	4,341 (0.6%)	11,439 (10.0%)
EDI Quintile, n (%)				
Q1 (least deprived)	66,389 (13.3%)	10,092 (24.9%)	118,691 (15.6%)	13,898 (12.1%)
Q2	78,578 (15.7%)	3,584 (8.9%)	112,593 (14.8%)	13,854 (12.1%)
Q3	74,384 (14.9%)	3,438 (8.5%)	111,310 (14.6%)	13,251 (11.6%)
Q4	137,234 (27.5%)	10,097 (24.9%)	221,903 (29.2%)	27,751 (24.2%)
Q5 (most deprived)	141,017 (28.2%)	12,791 (31.6%)	191,338 (25.2%)	34,286 (29.9%)
Missing	2,168 (0.4%)	482 (1.2%)	4,341 (0.6%)	11,439 (10.0%)
Number of chronic diseases, n (%)				
0	114,499 (22.9%)	11,877 (29.3%)	172,206 (22.7%)	56,005 (48.9%)
1	130,811 (26.2%)	10,636 (26.3%)	199,291 (26.2%)	24,891 (21.7%)
2	123,914 (24.8%)	9,162 (22.6%)	187,622 (24.7%)	17,238 (15.1%)
3	79,620 (15.9%)	5,507 (13.6%)	121,101 (15.9%)	9,755 (8.5%)
4	34,814 (7.0%)	2,274 (5.6%)	54,174 (7.1%)	4,406 (3.8%)
5+	16,112 (3.2%)	1,028 (2.5%)	25,782 (3.4%)	2,184 (1.9%)
Number of SARS-CoV-2 tests in 2021, n (%)				
0	390,166 (78.1%)	30,999 (76.6%)	597,080 (78.5%)	88,703 (77.5%)
1	66,129 (13.2%)	5,226 (12.9%)	95,874 (12.6%)	14,323 (12.5%)
2	23,741 (4.8%)	1,865 (4.6%)	34,980 (4.6%)	4,943 (4.3%)
3	8,557 (1.7%)	798 (2.0%)	12,593 (1.7%)	2,021 (1.8%)
4-9	9,835 (2.0%)	1,277 (3.2%)	16,586 (2.2%)	3,514 (3.1%)
10+	1,342 (0.3%)	319 (0.8%)	3,063 (0.4%)	975 (0.9%)
Any other vaccine, n (%)†	325,333 (65.1%)	18,503 (45.7%)	497,594 (65.5%)	21,684 (18.9%)

† received at least one of the following vaccines since 2016,influenza, pn23, pcv 7, 10 or 13
