# Supplementary material for: COVID-19 vaccine effectiveness against symptomatic SARS-CoV-2 infections, COVID-19 related hospitalizations and deaths, among individuals aged ≥65 years in Portugal: A cohort study based on data-linkage of national registries February-September 2021
Source: PLoS One. 2022 Sep 13;17(9):e0274008. doi: 10.1371/journal.pone.0274008 (PMC9469958; doi:10.1371/journal.pone.0274008)
Supplement: S2 Table — (PDF) [file pone.0274008.s004.pdf]

S2 Table. Demographic characteristics and vaccine status of cohort individuals aged 80 or more years old, Portugal, February–August 2021 (n = 470,025).

	Vaxzevria<br>8,509	Janssen<br>2,191	mRNA<br>435,322	Unvaccinated<br>24,003
Age (years), median (IQR)	82.0 (80.0 - 86.0)	85.0 (82.0 - 88.0)	84.0 (82.0 - 88.0)	86.0 (83.0 - 90.0)
Age group, (%)				
80-84	5,681 (66.8%)	1,078 (49.2%)	222,717 (51.2%)	8,997 (37.5%)
85-89	2,022 (23.8%)	732 (33.4%)	145,526 (33.4%)	8,137 (33.9%)
90-94	660 (7.8%)	304 (13.9%)	54,263 (12.5%)	4,879 (20.3%)
95+	146 (1.7%)	77 (3.5%)	12,816 (2.9%)	1,990 (8.3%)
Sex Male, n (%)	3,517 (41.3%)	769 (35.1%)	176,846 (40.6%)	8,645 (36.0%)
Region, n (%)				
Alentejo	1,308 (15.4%)	186 (8.5%)	24,081 (5.5%)	1,106 (4.6%)
Algarve	884 (10.4%)	313 (14.3%)	15,897 (3.7%)	1,412 (5.9%)
Centro	1,547 (18.2%)	519 (23.7%)	91,855 (21.1%)	4,627 (19.3%)
LVT	2,710 (31.8%)	699 (31.9%)	142,581 (32.8%)	7,969 (33.2%)
Norte	2,012 (23.6%)	460 (21.0%)	159,442 (36.6%)	8,095 (33.7%)
Missing	48 (0.6%)	14 (0.6%)	1,466 (0.3%)	794 (3.3%)
EDI Quintile, n (%)				
Q1 (least deprived)	1,078 (12.7%)	366 (16.7%)	76,030 (17.5%)	3,836 (16.0%)
Q2	1,032 (12.1%)	285 (13.0%)	68,094 (15.6%)	3,403 (14.2%)
Q3	1,112 (13.1%)	268 (12.2%)	66,021 (15.2%)	3,582 (14.9%)
Q4	2,294 (27.0%)	519 (23.7%)	120,749 (27.7%)	6,312 (26.3%)
Q5 (most deprived)	2,945 (34.6%)	739 (33.7%)	102,962 (23.7%)	6,076 (25.3%)
Missing	48 (0.6%)	14 (0.6%)	1,466 (0.3%)	794 (3.3%)
Number of chronic diseases, n (%)				
0	956 (11.2%)	313 (14.3%)	44,531 (10.2%)	8,927 (37.2%)
1	1,529 (18.0%)	382 (17.4%)	82,962 (19.1%)	3,687 (15.4%)
2	2,024 (23.8%)	523 (23.9%)	112,314 (25.8%)	4,258 (17.7%)
3	1,885 (22.2%)	471 (21.5%)	96,833 (22.2%)	3,620 (15.1%)
4	1,207 (14.2%)	288 (13.1%)	58,377 (13.4%)	2,002 (8.3%)
5+	908 (10.7%)	214 (9.8%)	40,305 (9.3%)	1,509 (6.3%)
Number of SARS-CoV-2 tests in 2021, n (%)				
0	5,651 (66.4%)	1,330 (60.7%)	327,670 (75.3%)	15,614 (65.1%)
1	1,304 (15.3%)	329 (15.0%)	52,314 (12.0%)	3,110 (13.0%)
2	624 (7.3%)	165 (7.5%)	22,336 (5.1%)	1,719 (7.2%)
3	330 (3.9%)	86 (3.9%)	11,110 (2.6%)	1,033 (4.3%)
4-9	524 (6.2%)	230 (10.5%)	19,170 (4.4%)	2,218 (9.2%)
10+	76 (0.9%)	51 (2.3%)	2,722 (0.6%)	309 (1.3%)
Any other vaccine, n (%)†	8,047 (94.6%)	1,984 (90.6%)	420,208 (96.5%)	19,865 (82.8%)

† received at least one of the following vaccines since 2016,influenza, pn23, pcv 7, 10 or 13
